# The Viability of Single Cancer Cells after Exposure to Hydrodynamic Shear Stresses in a Spiral Microchannel: A Canine Cutaneous Mast Cell Tumor Model

**DOI:** 10.3390/mi9010009

**Published:** 2017-12-28

**Authors:** Dettachai Ketpun, Achariya Sailasuta, Thammawit Suwannaphan, Sudchaya Bhanpattanakul, Alongkorn Pimpin, Werayut Srituravanich, Witsaroot Sripumkhai, Wutthinan Jeamsaksiri, Prapruddee Piyaviriyakul

**Affiliations:** 1 Biochemistry Unit, Department of Physiology, Faculty of Veterinary Science, Chulalongkorn University, Bangkok 10330, Thailand; dketpun@ntu.edu.sg; 2 Companion Animal Cancer-Research Unit (CAC-RU), Department of Pathology, Faculty of Veterinary Science, Chulalongkorn University, Bangkok 10330, Thailand; achariya.sa@chula.ac.th (A.S.); Sudchaya.Bha@student.chula.ac.th (S.B.); alongkorn.p@chula.ac.th (A.P.); werayut.s@chula.ac.th (W.S.); 3 Research Fellow in Biomedical Engineering, School of Mechanical and Aerospace Engineering, Nanyang Technological University, Singapore 639798, Singapore; 4 Department of Mechanical Engineering, Faculty of Engineering, Chulalongkorn University, Bangkok 10330, Thailand; Thammawit.Su@student.chula.ac.th; 5 Thai Microelectronic Centre, Ministry of Science and Technology, Chachoengsao 24000, Thailand; witsaroot.sripumkhai@nectec.or.th (W.S.); wutthinan.jeamsaksiri@nectec.or.th (W.J.)

**Keywords:** hydrodynamic shear stress, microfluidic, mast cell tumor, sorting, spiral microchannel, viability

## Abstract

Our laboratory has the fundamental responsibility to study cancer stem cells (CSC) in various models of human and animal neoplasms. However, the major impediments that spike our accomplishment are the lack of universal biomarkers and cellular heterogeneity. To cope with these restrictions, we have tried to apply the concept of single cell analysis, which has hitherto been recommended throughout the world as an imperative solution pack for resolving such dilemmas. Accordingly, our first step was to utilize a predesigned spiral microchannel fabricated by our laboratory to perform size-based single cell separation using mast cell tumor (MCT) cells as a model. However, the impact of hydrodynamic shear stresses (HSS) on mechanical cell injury and viability in a spiral microchannel has not been fully investigated so far. Intuitively, our computational fluid dynamics (CFD) simulation has strongly revealed the formations of fluid shear stress (FSS) and extensional fluid stress (EFS) in the sorting system. The panel of biomedical assays has also disclosed cell degeneration and necrosis in the model. Therefore, we have herein reported the combinatorically detrimental effect of FSS and EFS on the viability of MCT cells after sorting in our spiral microchannel, with discussion on the possibly pathogenic mechanisms of HSS-induced cell injury in the study model.

## 1. Introductory Background

A neoplasm is composed of heterogeneous cell subpopulations [1,2] in which one cell species, referred to as cancer stem cells (CSCs), plays a central role as the headwater of oncogenesis. As a cancer stem cell hypothesis, CSCs always possess the disparate biological property called stemness. They are immortal and responsible for intra-neoplastic heterogeneity [3,4]. Therefore, the isolation of CSCs dwelling in neoplasms is an indispensable process for studying their intricate biology. Absolutely, the outright comprehension of CSC biology will pave us the way to establish the most suitably targeted therapy for disease annihilation in the upcoming future. However, the major roadblocks are cellular heterogeneity itself and that currently no reliably universal biomarkers are available to definitely identify CSCs.

Theoretically, neoplastic cells in a given neoplasm are not biophysically identical, particularly their diverged sizes. Fortunately, many studies have potentially suggested that the sizes of putative cancer stem cells in a given cancer may be smaller than 10 μm (average at 5–7 μm); meanwhile, the major constituent cells—terminally differentiated cancer cells—are frequently larger than 10 μm [5]. Thus, the usage of size-based cell segregation may perceivably be the most convenient way to harvest viably putative CSCs without any labelling process. Nevertheless, single cell analysis has hitherto been recommended worldwide as a solution pack for demolishing the impact of intra-neoplastic heterogeneity [6,7,8]. Notwithstanding, the scantiness of a trusty method for label-free single cell isolation is still the critical hitch. There are many contemporary research tools, such as fluorescence-activated cell sorting (FACS), magnetic-activated cell sorting (MAC), electrophoresis, and laser microdissection (LMD), which can enable cell biologists to achieve these aims. However, almost all of them are label-dependent. They also require prolonged and intricate sample preparation that is harmful to studied cells [9,10].

A blessing in disguise, microfluidics has recently been developed and introduced throughout the world as an attractive means for label-free single cell separation [11]. This innovatively integrative science and engineering technology is capable of handling microparticles, including cells in a downscale microchannel, precisely [1,12]. During the last decade, both active and passive microfluidics have been used for this purpose so far [13,14]. Basically, active microfluidics, including magnetophoresis, acoustophoresis, and dielectrophoresis, require external force fields to stabilize their performances. In the meantime, the passive regimes, such as deterministic lateral displacement (DLD) and centrifugal (gravitational) sedimentation, always use the geometry of the microfluidic microchannel and their inherited hydrodynamics to manipulate the cells.

Even though the active processes are precisely controllable and somewhat sensitive, their applications are frequently unfavorable because of their low-throughput performance and the external force field application and multiplex auxiliary system required. These might result in an increased complexity of device fabrication. Moreover, the residential time of the sorted cells in the microchannel is usually prolonged. Thence, they can consistently accumulate more stresses from the external fields [15,16]. On the other hand, the use of the internal hydraulic properties of fluids in passive microfluidics is now more admirable according to its high-throughput rate. Furthermore, the processes are anticipated not to be hurtful to the cells of interest because no external forces are applied [17].

For passive microfluidic cell separation, an inertial cell focusing on secondary flow fields, in particular Dean Vortices, is the most usable methodology. A plethora of scientific evidence has demonstrated the successfulness of its application worldwide. For instance, Kuntaegowdanahalli and colleagues employed an Archimedean spiral microchannel for sorting 15 μm SH-SY5Y-neuroblastoma cells from 7 μm C6-rat-glioma cells [15]. Additionally, Hur and his team also utilized a similar configuration of a spiral microchannel to segregate human white blood cells (WBCs) from red blood cells (RBCs). The other intriguing achievement was the separation of bacteria contaminating diluted human blood by Wu’s research team [18], as well as the sorting of human platelets from other blood components [9].

In addition, our preliminary studies have substantially demonstrated the efficacy of our predesigned Archimedean spiral microchannel in the separation of multiple-sized polystyrene microspheres, intact canine blood cells and feline leukemic cells at the optimal flow rate of 1 mL·min^−1^ [19,20], and naturally-buoyant canine cutaneous mast cell tumor (MCT) cells harvested by fine-needle aspiration (FNA). In that study, the results indicated that our microdevice could efficiently isolate 15–25 μm terminally differentiated MCT cells from the smaller contaminated cells, including putative MCT cancer stem cells (<7 μm), which were unfocused. The sorted MCT cells were substantially harvested at the outlet III of the microdevice. Meanwhile, unfocused contaminants dispersed throughout the device and could be collected from all outlets [21].

Although most of the studies would indicate the powerful performance of a spiral microchannel in inertial microfluidic cell separation, they have almost always emphasized the effects of curvilinear geometry and the physics of fluid flow on focusing resolution instead of the underlying dreadful factors [22], especially hydrodynamic shear stresses (HSS). Therefore, information on cell viability under inertial microfluidic cell sorting is still scarce, even though the measuring of cell viability is the essential consideration in all aspects of biomedical engineering research [23,24,25].

Because of the lack of cell walls, all mammalian cells under a passive microfluidic platform are prone to be mechanically damaged after experiencing hydrodynamic shear stresses (HSS) [26,27,28]. However, fluid flow in a microsystem inevitably produces hydrodynamic shear stresses, especially at a high-throughput rate. Therefore, they could forthwith induce cell degeneration and necrosis. Principally, in a straight microchannel, both hydrodynamic shear components—fluid shear stress (FSS) and extensional fluid stress (EFS)—are generated when the velocity of flow has been sharply changed. FSS is the velocity gradient of fluid flows between the axis and the boundary. In the meantime, EFS usually occurs along the flow axis when a transection of the microchannel instantaneously alters from the wider to the narrower [29]. However, the formations of both HSS components and their impacts on cell viability in a spiral microchannel have not been described so far.

For a spiral microchannel, several studies have suggested that the viability of a sorted cell is greater than 80% [15,30]. However, the effects of hydrodynamic shear stresses were not stringently evaluated in those experiments. In accordance with one of our previous studies, the result has substantially unveiled that the viability of most canine leukocytes was also greater than 70% after sorting [31]. The computational fluid dynamics (CFD) had revealed the formations of hydrodynamic shear stresses in our sorting system at the flow rate of 1 mL·min^−1^ [20].

Fundamentally, canine cutaneous mast cell tumors (MCT) are a member of canine round cell tumors. It implies that their morphological feature is uniquely round and close to the ideal cells or polystyrene microbeads commonly engaged for testing the theoretical functionality of a spiral microchannel. However, cellular pleomorphism may occur in the high-grade [32,33]. Therefore, the utility of MCT cells makes them reasonable for use in an appropriate experimental setup and result interpretation of cells sorted with the spiral microchannel.

Besides, our laboratory has currently been endeavoring to establish a label-free passive microfluidic platform which is capable of tracking and isolating putative CSCs depending on their sizes in various human and animal study models. Furthermore, based on our supposition, the platform must (1) be high-throughput and highly sensitive [11,34,35], (2) not be harmful to analyzed cells, and (3) furnish statistically meaningful information on the biology of studied cells. Hence, the vitality of the sorted cells is the primary concern to assure the safety of the sorting system.

By way of introduction, there is a proclivity to isolate putative cancer stem cells from their non-CSC counterparts based on the diversity of cell sizes with the spiral microchannel, even though there are the formations of hydrodynamic shear stresses in the sorting system. In addition, most studies have claimed that they have no any deleterious effect on normal mammalian cells after sorting. However, as neoplastic cells are preposterous cells, their external and internal mechanical morphology may be different from normal cells. Thus, the fatal effect of hydrodynamic shear stresses on neoplastic cells is still dubious whenever inertial microfluidic cell sorting is applied to these cell species.

Taken together, the vitality of the sorted neoplastic cells is the primary concern, while the hydrodynamic shear stresses in a spiral microchannel are inescapable. However, there is a need to segregate single putative CSCs from non-CSCs by their sizes with the spiral microchannel. Thence, to assure the safety of the sorting system, the aim of this study was to demonstrate systematically the viability of the sorted MCT cells after being exposed to hydrodynamic shear stresses in our predesigned Archimedean spiral microchannel. We speculated that the study outcome will be beneficial for everyone who needs to improve the functionality of their spiral microchannel before the microdevice will become a primary method for isolating single putative cancer stem cells in the coming years.

## 2. Materials and Methods

### 2.1. Theoretical Background for Spiral Microchannel Design

Fundamentally, inertial focusing under microfluidic platforms utilizes the intrinsic hydrodynamic effect of one or more fluid sheaths flowing around microparticulates, including cells, for aligning them into two-dimensional (2D) bands or three-dimensional (3D) single streamlines [17,36]. For a rectangular spiral microchannel, cell focusing emerges when the fluid medium has been turning the curves. By this, two counter-rotating secondary flows called Dean Vortices originate at the top and the bottom halves of microchannel cross-sections [37]. In accordance with the centrifugal effect of the Dean Vortices, two additional counter-rotating components referred to as Dean Drag Forces (F_D_) will have been awakened [9]. They further push cells across the main flow streams to the inner sidewalls of the microchannel in the opposite directions. The magnitude of these forces is governed by known parameters as follows: F_D_ = 3πμU_Dean_a_p_ or 5.4 × 10^−4^πμDe^1.63^a_p_; where μ, U_Dean_, a_p_, and De are the viscosity of the fluid media, the Dean velocity (U_Dean_ = 1.8 × 10^−4^ De^1.63^), the diameter of each microparticle, and the Dean Number, respectively.

When the cells have been moving closer to the inner walls, they are counteracted by inertial repulsion forces (inertial lift forces or F_L_) generated from the inner walls. This brings about the backward migration of the cells to the microchannel centre. Basically, the inertial lift forces are equated with the following parameters: F_L_ = ρG^2^C_L_a_p_^4^; where ρ, G, and C_L_ are the fluid density, shear rate, and lift coefficient, respectively [15]. At a certain lateral displacement from the inner walls, individual cells with the same diameters are paused because F_D_ and F_L_ are equipoising. It causes the cells to entrain into a focusing streamline. Since two counterbalanced forces and cell diameters handle the stream focusing, larger cells will reach their stabilized locations closer to the inner walls than smaller cells [14,15]. Additionally, the inertial focusing is determined by the confinement ratio (ω) between the diameter of the microparticles (a_p_) and the microchannel height (H), expressed by ω = a_p_/H [14]. Basically, it must be equal to or greater than 0.07 [15,38,39].

### 2.2. Microdevice Fabrication and Instrumentation

Briefly, the microdevice was fabricated with the standard photoresist-soft-lithography. Stepwise, the silicon mold was constructed by transferring the tracery of the microchannel onto a 6-inch silicon wafer by exposure to UV through the photoresist mask. The wafer was chemically etched. Further, liquid elastomer, polymethysiloxane (PDMS), was poured on the mold and was allowed to cool down. The PDMS replica was peeled off and bounded on a 5 cm × 7.5 cm glass slide with oxygen plasma. The inlets and the outlets of the microdevice were drilled using a metal punch and bonded to silicone tubes glued by liquid PDMS.

The instrumentation of the microdevice initiated with the connection of the inlets to the feeding conduits: one for cell suspension and the other for the fluid buffer. Each feeding conduit was systematically composed of four separated portions: a 3 mL plastic syringe with a lure tip (Nipro^TM^, Osaka, Japan), a 20G3/4 intravenous catheter (Nipro^TM^), a 20 cm silicone tube (Cole-Parmer^TM^, Vernon Hills, IL, USA), and a 21G1 metal connector. Both syringes were simultaneously empowered by a two-port programmable automatic syringe pump (F-100, Chemyx, Stafford, TA, USA). The optimal flow rate used in this study was at 1 mL·min^−1^ based upon the previous studies [19,20,21]. Data acquisitions were done with reverse light microscopy.

### 2.3. Computational Emulation of Fluid Shear Stress and Extensional Flow Stress

In the study, we systematically operated computerized simulations to predict the formations of two main hydrodynamic stress components: fluid shear stress (FSS) and extensional flow stress (EFS). The simulation was performed using a modeling software, COMSOL Multiphysics^®^ version 5.3 (COMSOL, Los Angelis, CA, USA), under the finite element method. The predilection parameters of the fluid were selected from the software; meanwhile, the equations for the simulation were manually determined as following; FSS = μ(∂v/∂z + ∂w/∂y) and EFS = 3μ(∂u/∂x). The computational fluid dynamics (CFD) were performed at the first loop of the spiral microchannel. The simulations were grid-dependent. The spiral loop was modeled with a 5 × 10^6^ controlling meshwork. Moreover, the fluid medium was supposed to be incompressible Newtonian fluid with a batch of physicochemical properties of density at 1000 kg·m^−3^ and a dynamic viscosity of 1.003 × 10^−3^ kg·m^−1^·s^−1^ at 20 °C. A symmetrically non-slipped boundary condition was used for establishing the flow condition. The flow rate was 1 mL·min^−1^, and the characteristic of the flow was uniformity. The simulations were performed at the final loop of the spiral microchannel. The magnitudes of FSS and EFS were calculated by the software at the outlet and at the curvatures of the spiral microchannel, respectively [31].

### 2.4. Single MCT Cell Isolation

To reduce the bias on cell vulnerability, freshly single MCT cells were noninvasively isolated and harvested from MCT masses removed by operative interventions at the Faculty of Veterinary Science, Chulalongkorn University, during 2015 to early 2016. Stepwise, one gram of each lean MCT tissue was chopped into tiny cubes (approximately 2 mm × 2 mm × 2 mm). Each diminutive tissue specimen was further collected in a 2 mL centrifuge tube and was trypsinized (200 μL of 0.025% Trypsin and 0.01% EDTA in 500 μL PBS) at 38 °C for 20 min. The reaction was then terminated with 1 mL of 2% fetal calf serum (FCS). Undigested tissue remnant was filtered out via a 30 μm cell strainer. The cell flow-through of each was pooled together into a 15 mL centrifuge tube and was centrifuged at 885 RCF (Eppendorf 5430, Eppendorf AG, Mississauga, ON, Canada) for 5 min. The cell pellet was then washed with phosphate-buffered saline (PBS) and was centrifuged at 885 relative centrifugal force (RCF) for 5 min at room temperature twice. Finally, single MCT cells were re-suspended in PBS. In addition, one-third of each mass was sent to the Department of Pathology, Faculty of Veterinary Science, Chulalongkorn University, for routine histopathology.

### 2.5. Systematic Biomedical Assays for Mast Cell Tumor (MCT) Cell Viability

**Figure 1:**
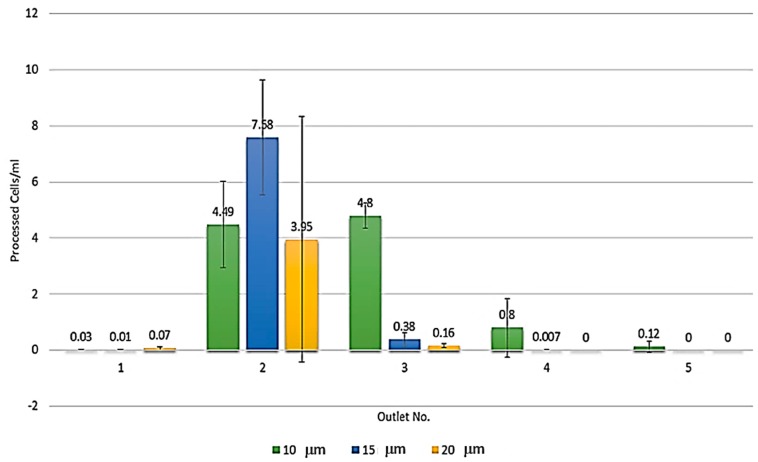
The histogram plotted from the preliminary data illustrates the tendency of mast cell tumor (MCT) cell focusing and distribution in our predesigned spiral microchannel. Most of the larger cells sized from 10 μm were focused and distributed through the outlets II and III of the microdevice. In contrast to small MCT cells, they were unfocused and dispersed through the outlets I to V. Notably, the sizes of individual MCT cells from each outlet were measured by fluorescence-activated cell sorting (FACS) size-based quantification [21].

Based on our preliminary study, the result has revealed the distributive trend of MCT cells after sorting as shown in Figure 1. Briefly, single MCT cells sized from 10 μm were substantially focused and mainly distributed through the outlets II and III of the microdevice. Meanwhile, smaller cells were unfocused based on their confinement ratios, which were less than 0.07. Notably, no MCT cells dispersed through the outlets VI to X.

MCT cells from each outlet were then obtained and used for systemic viability assays. The appraisal criteria were composed of (1) cell morphology, (2) cell membrane integrity, and (3) cell functionality as previously described in the literature [25]. It is noteworthy that a panel of biomedical investigations was also performed in unsorted MCT cells as the reference for result comparisons.

#### 2.5.1. Morphological Investigation

MCT cells were re-suspended with 1 mL PBS. Further, 35 μL of the cell suspension was smeared onto a clean glass slide. The specimen was stabilized with a cover slip. Cell configuration was visualized under plain light microscopy at the high power field (40×), and data acquisition was performed with the following interpretative benchmarks.

Morphometrically, the cell morphology index (CMI), the ratio of the width and the length of MCT cells, was measured. This evaluative parameter principally indicates the ability of non-degenerated MCT cells to cope with mechanical induction factors, including hydrodynamic shear stresses, by allowing them to be elongated and recoiled. Ideally, the CMI is equal to one in normal MCT cells; meanwhile, the index is zero (unmeasured) when MCT cells are necrotized despite cell wreckage. In the case of cell deformation (benign degeneration), the index would fall into the range in between zero and one.

#### 2.5.2. Assessments of Cell Membrane Disintegration

To verify membrane disintegration, we performed scanning electron microscopy (SEM, JEOL Ltd., Tokyo, Japan), a measurement of DNA content leakage, and a trypan blue exclusion assay under their standard protocols to test membrane discontinuity.

Scanning electron microscopy (SEM) was performed under a recommended protocol previously used for the study of chicken leukocytes. Briefly, single MCT cells were washed twice and re-suspended in 1 mL Hank’s balanced salt solution (HBSS). They were fixed in 1.2% glutaraldehyde in 0.1 M sodium cacodylate (CAC) buffer (pH 7.3) for 1 h and washed twice in 0.16 M CAC buffer (pH = 7.3). They were further post-fixed in 1% osmium tetroxide (OsO_4_) in 0.1 M CAC buffer (pH = 7.3) for 1 h. The cells were then serially dehydrated by graded ethanol (25%, 50%, 75%, 95%, and 100% for 10 min of each), a series of three mixtures of ethanol-iso-amyl acetate at the ratio of 3:1, 1:1, and 1:3, and pure iso-amyl acetate. Several drops of cell suspension were transferred onto 10mm aluminum membrane dishes and allowed to settle for 2 min. Finally, the cells were dried in CO_2_ in a critical point dryer, and the specimen dishes were attached to aluminum stubs by carbon–propanol glue and coated with gold in a sputtering device. The MCT cells were examined with a Scanning Electron Microscope (JSM-6610LV, JEOL Ltd., Tokyo, Japan) at the acceleration voltage of 15 kV [40]. The positivity was identified by cell membranes that are perforated or ruptured. In addition, their surface proteins are also deleted or collapsed. On the other hand, the cell membranes of non-degenerated MCT cells are integrated. Their external surface proteins are also normal in orientation.

Leaky DNA owing to cell rupture or an increment increase in membrane permeability was measured with standard spectrophotometry. Briefly, MCT cell suspension from each outlet was harvested after sorting and centrifuged (Eppendorf 5430, Eppendorf AG) at 394 RCF for 5 min. The bias of protein effect on optical density (OD) was reduced by purifying DNA in the supernatant using a commercial DNA extraction kit (Mobio, Carlsbad, CA, USA). Stepwise, the supernatant was transferred onto a silica membrane-bounded collection tube and centrifuged at 10,000 RCF for 1 min. DNA on the silica membrane was washed with 500 μL TD-2 washing solution and centrifuged at 10,000 RCF for 1 min twice. Ultimately, DNA was eluted by incubating with 50 μL TD-3 eluting solution and centrifuging at 10,000 RCF for 1 min. The purified DNA solution was then diluted with deionized (DI) water at the ratio of 1:100. The optical density (OD_260_) of DNA was measured by a spectrophotometer at the wavelength of 260 nm (A_260_). The DNA concentration was calculated back by the following equation: dsDNA concentration = 50 μg/mL × OD_260_ × dilution factor.

For cell permeability, a trypan blue exclusion assay was utilized. Stepwise, 300 μL MCT cell suspension was aliquoted to a new 2 mL collection tube. The cells were incubated by 500 μL of 0.4% Trypan blue (Hyclone^TM^, GE Healthcare Life Sciences, Chicago, IL, USA) in 200 μL PBS at room temperature for 5 min in a dark chamber. The cells were twice washed with PBS and centrifuged at 885 RCF for 10 min.

The permeability was evaluated to distinguish living MCT cells from dead cells. MCT cells which undergo necrosis will stain blue and healthy MCT cells stain negative. The concentration of viable cells was also enumerated as the percentage of viable cells using hemocytometry [41].

#### 2.5.3. Evaluation of Functional Viability

In this step, we examined viable cell functionality with a tetramethylrhodamine-methyl ester-perchlorate (TMRM) cationic probe (Thermo Fisher Scientific, Waltham, MA, USA). Briefly, 200 μL of MCT cells in PBS was incubated with 1 mL of 10 μM TMRM in PBS at 25 °C for 30 min. Excessive dyes were flushed out from the suspension by PBS. The fluorescent signals were acquired under fluorescent microscopy with a tetramethylrhodamine (TRITC) filter. Basically, active mitochondria in healthy MCT cells usually stain with TMRM; meanwhile, only cortical mitochondria are positive for TMRM in case of degenerated and necrotic MCT cells.

## 3. Results

### 3.1. Microdevice Architecture

**Figure 2:**
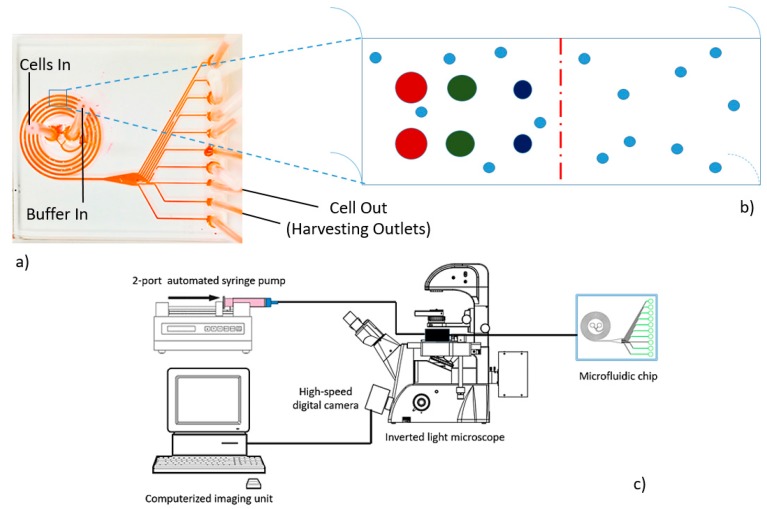
The configuration of the predesigned Archimedean spiral microchannel used in this study. (**a**) The outward appearance of the microdevice consisting of two symmetrical inlets (for cells and buffer, respectively) and a port of ten asymmetrical outlets for sorted cell collections. (**b**) The cross-sectional outline of the curved rectangle microchannel exhibits the predicted lateral displacements of MCT cell focusing in the spiral microchannel. (**c**) The instrumentation diagram of the sorting system employed in this study. The system is mainly composed of a two-port automatic syringe pump, an inverted light microscope connected to a high-speed digital camera, and a computerized imaging unit.

Under standard photoresist-soft-lithography, we had successfully fabricated our predesigned Archimedean spiral microchannel (ASM) with polydimethylsiloxane (PDMS). The geometrical configuration of the microdevice consisted of a five-turned curvilinear rectangle microchannel with a constant interspace between two adjacent loops of 500 μm. At the cross-section, the dimension of the microchannel was 500 μm in width and 130 μm in height. The instrumentation is schematically illustrated in Figure 2c. The microdevice connected to a port of a dual-feeding inlet. The first one was for delivering the cell suspension; meanwhile, the second was for the control buffer. The exit slot was a single 720-μm-long conical microchannel connecting to a port of ten asymmetrical outflow tracts (Figure 2a).

### 3.2. Single MCT Cell Isolation and Morphological Characterization of Unsorted MCT Cells

**Figure 3:**
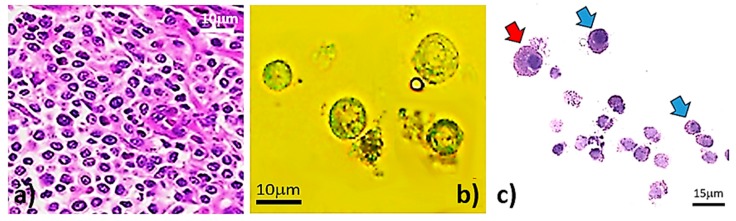
Normal configurations of unsorted MCT cells. (**a**) Histopathology of MCT hematoxylin and eosin (H&E). It is noteworthy to address that the arrangement of MCT cells was sheet-like surrounded by thin fibrovascular stroma. Almost cells were round and monomorphic. Their cytoplasm was fully filled with metachromatic granules. The nuclei were round with condensed hyperchromatic chromatins. Mitotic cells were moderately observed in all studied specimens. (**b**) The normal configuration of single MCT cells isolated by trypsinization under plain light microscopy. (**c**) The external appearances with eosin-methylene blue (EMB) staining. The result verified that the MCT cells were round to slightly oval cells with a basophilic cytoplasm. Fundamentally, their cytoplasm contained finely basophilic and/or clearly glistening vacuolar granules. Moreover, their nuclei were basophilically hyperchromic.

Principally, unsorted MCT cells were non-degenerated cells. Histopathological sections disclosed that all neoplastic masses in this study are canine cutaneous mast cell tumors (Figure 3a). By trypsinization, we successfully harvested single MCT cells from the masses. Further evaluation under plain light microscopy obviously revealed that the major population of trypsinized cells was composed of single MCT cells.

Cytologically, non-degenerated MCT cells are round cells (Figure 3b); however, they are slightly oval in such circumstances as well. Their cytoplasm is usually transparent and filled with variably sized refractive granules, which contain major preformed-water-soluble chemomediators in cytoplasmic granules, such as histamine, serotonin, sulfated glycosaminoglycan, proteoglycan heparin, and chondroitin sulfate-E [42,43,44,45]. The nuclei are usually single, large, and round to trivially oval in shape with a prominent single nucleoli. Moreover, they may be concentric or eccentric nuclei.

However, some MCT cells may contain binuclei or even polynuclei as well. The nucleus-to-cytoplasm (N/C) ratio varies from 1:1 to 2:1. Individually, the sizes are often varied from 6 to 15 μm; however, aberrantly non-degenerative MCT cells may be larger than 15 μm [44,46]. With the eosin-methylene blue (EMB) staining, the result unveiled a group of round to slightly oval MCT cells. The cytoplasm was basophilic, containing finely basophilic and/or clearly glistening vacuolar granules; meanwhile, their nuclei were hyperchromic (Figure 3c).

### 3.3. Systematic Biomedical Engineering Assays of the Viability of the Sorted MCT Cells

**Figure 4:**
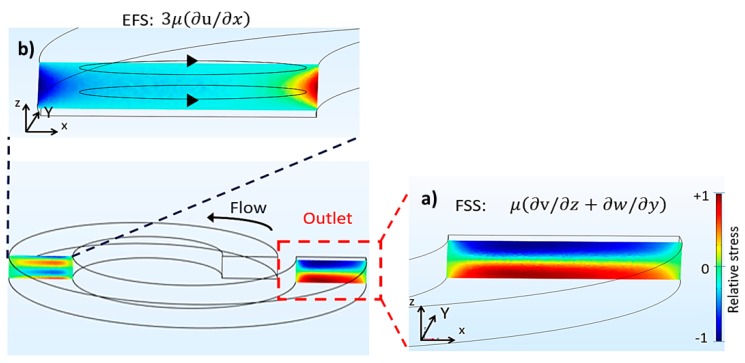
The formations of two major hydrodynamic shear stresses: fluid shear stress (FSS) and extensional fluid stress (EFS) in our predesigned Archimedean spiral microchannel (ASM) conducing to cell degeneration and necrosis of the sorted MCT cells. (**a**) The fluid shear stress was mainly maximal at the upper and lower perimeters, but in opposite directions. (**b**) The extensional fluid stress maximally took place at the lesser curvature of the ASM.

Step by step, our CFD distinctly exhibited the rising up of fluid shear stress (FSS) and extensional fluid stress (EFS) in our predesigned spiral microchannel. At the selected flow rate, the computational simulation exhibited the generation of FSS at the upper and the lower perimeters of the microchannel (Figure 4a). Meanwhile, EFS originated both at the lesser and greater curvatures (relative positive and negative EFS, respectively) caused by the extension and shrinkage of fluids (Figure 4b). Accordingly, an EFS gradient had taken place, resulting in the formation of a virtual hydrodynamic wall. Finally, it led to a sudden change of microchannel transection. Remarkably, the extensional flow stress (EFS) was obviously invoked in the square manner of FSS. The maximum amplitudes of FSS and EFS computed by the software were approximately 20 Pa and 10 Pa, respectively.

**Figure 5:**
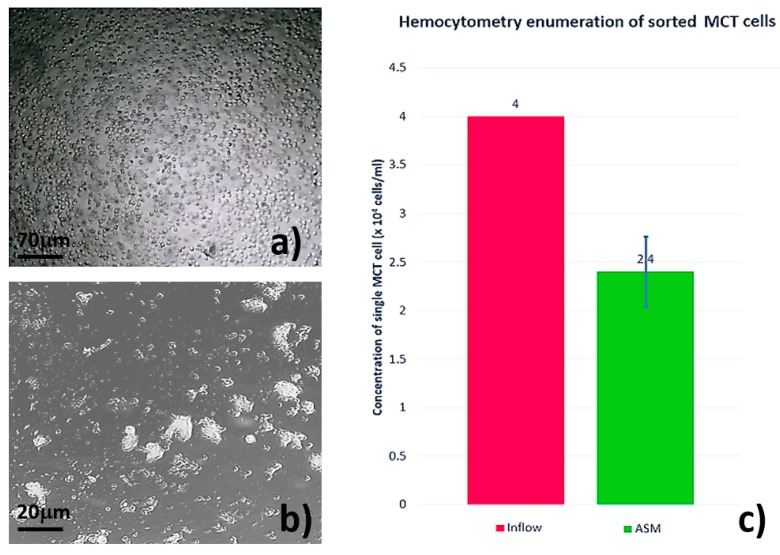
The aftermath of hydrodynamic shear stresses on the cell viability of the sorted MCT cells. (**a**) The morphology of MCT cells before sorting indicated that unsorted MCT cells were round and discrete. The concentration of viable MCT cells was plentiful. (**b**) The micrograph exhibits cellular attrition in the system. The sorted MCT cells were deformed or necrotized. Notably, their morphological features were changed, for example elongation and severe swelling. Cellular destruction and the protrusion of cytoplasmic granules were commonly seen in the field. (**c**) Likewise, the cellular enumeration with the hemocytometer overtly confirmed a reduction of viably sorted MCT cells, in which the cellular concentration after sorting was down to 2.4 × 10^4^ cells/mL (green bar).

In the study outcome, plain light microscopy uncovered cell vanishment in our sorting system (Figure 5). Initially, the concentration of unsorted MCT cells was abundant at 4 × 10^4^ cells/mL. Obviously, the cells were morphologically round and solely separated. The cellular contour was normal in range as previously described (Figure 5a). After sorting, the concentration of MCT cells was reduced. The morphology of the sorted cells suggested grievous cell attrition in the system. They underwent deformation and/or malignant degeneration (necrosis). Explicitly, most of the sorted cells were severely swollen, and many cells were elongated. Their shapes were frequently distorted. Cellular laceration and the protrusion of cytoplasmic granules were commonly observed, indicating a sudden onset of cell membrane disintegration (Figure 5b). Likewise, the cellular enumeration with the hemocytometer overtly confirmed a reduction of viably sorted MCT cells, in which the cellular concentration after sorting was down to 2.4 × 10^4^ cells/mL as summarized in the histogram of Figure 5c.

**Figure 6:**
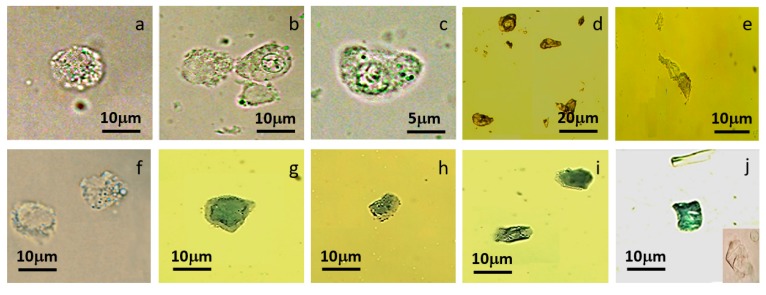
A panel of MCT cell micrographs after sorting through our predesigned spiral microchannel. (**a**) The external appearance of a viable sorted MCT cell was similar to fresh, non-degenerated MCT cells before sorting (Figure 4b). Remarkably, the cell morphology index (CMI) was equal to one according to the round shape. Nevertheless, all viable MCT cells were negative for trypan blue. (**b**,**c**) Plenty of the sorted MCT cells benignly degenerated. Their normal configurations were initially elongated or aberrant, with the escalated CMI varying from 0.22 (severe generation) to 0.625 (mild degeneration). (**d**,**e**) In the case of cell necrosis, sorted MCT cells were severely elongated or unidentifiable, bringing about debris formation. Nuclear dislocation or vanishment was stringently observed in these cells as well. Their CMIs were often lower than 0.22 or down to zero owing to their bizarre morphology. (**f**) The figure exhibits the trypan positivity of two benignly degenerated MCT cells after sorting, although their external features would be similar to non-degenerated MCT cells. (**g**,**h**) The figures depict two deformed cells fully stained with trypan blue. (**i**) The trypan blue exclusion assay identifies two malignantly degenerated MCT cells. The right upper cell was severely deformed and their nuclear-to-cytoplasm ratio was increased; meanwhile, the left lower cell was necrotized. Its morphology was distorted from that of normal MCT cells. Moreover, the nucleus (at the centre) deemed to be sloughed. (**j**) This figure depicts the positivity of trypan blue staining in MCT debris. The right lower inset illustrates the characteristics of MCT debris under plain light microscopy.

As predicted, the light microscopy substantially indicated cell degeneration and necrosis of MCT cells after inertial sorting with our predesigned spiral microchannel. Remarkably, the configurations of viably sorted MCT cells were intact with their CMI at 1. Moreover, they were negative for the trypan blue exclusion assay (Figure 6a) as in viably unsorted MCT cells (Figure 4b). Despite the results, a number of the sorted MCT cells were benignly degenerated with or without cell deformation. Obviously, some cells had lost their normal contours; meanwhile, others initially underwent elongation. However, cytoplasms, as well as cytoplasmic granules, were often intact in size and shape. Their nuclei were principally in situ. Nevertheless, their plasma membranes seemed to be unremitting (Figure 6b,c). In the case of malignant degeneration (necrosis), sorted MCT cells were severely elongated or their configurations were unidentifiable from time to time (Figure 6d). Cell wreckage was strongly observed in sorted MCT cells, with severe mechanical injuries resulting in cell debris formation. Nuclear dislocation or sloughing was a harshly necrotic figure of these cells as well (Figure 6e). The escalation of the cell morphology index (CMI) also revealed that the average CMI of deformed cells varied from 0.22 (severe) to 0.625 (mild). In the meantime, the CMI of necrotic MCT cells was zero (unmeasurable) because of their acellular configuration.

**Figure 7:**
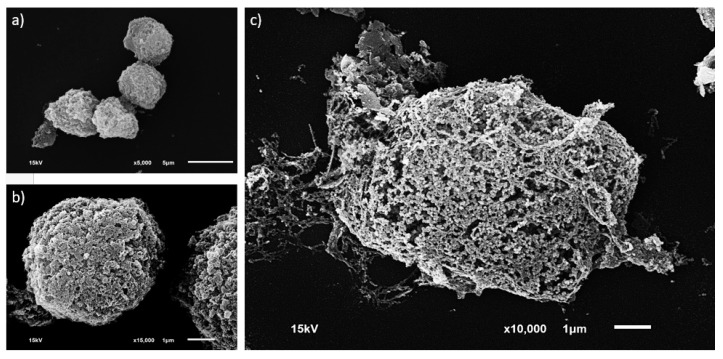
Scanning electron microscopy (SEM). (**a**) The figure illustrates a group of viably non-degenerated MCT cells harvested after sorting in this study. Most cells were round and aggregated. (**b**) The external characteristics of a viable MCT cell from above at the higher magnification. Notably, its plasma membrane was unremitting and its integral proteins on the external surface were kept normal. (**c**) Obviously, a single MCT cell underwent necrosis and was deformed. The cell membrane was disintegrated causing membrane perforation, and the membrane surface proteins were depleted.

Regarding the trypan blue exclusion assay, some MCT cells contained cytoplasmic granules, which were remarkably positive with trypan blue, even though the configurations of MCT cells were unchanged (Figure 6f). Intuitively, a number of the sorted MCT cells positively stained with trypan blue, even though their external appearances were normal (Figure 6g). Therefore, the final number of viable MCT cells was finally down to 2.4 cells/mL collated to non-trypan blue enumeration (Figure 7). Undoubtedly, most degenerated, deformed, and/or necrotized MCT cells after sorting were stringently positive with the trypan blue assessment (Figure 6h,i). Their cytoplasmic volumes and contents were remarkably receded, causing the accruement of the N/C ratio in such circumstances (Figure 6i). Cell debris also positively stained with trypan blue. They were characterized by membrane-bounded cytoplasm remnants of the lacerated cells. Additionally, the cytoplasmic granules and the nuclei were almost always absent (Figure 6j). Although trypan blue positivity would indicate the discontinuity of plasma membranes, such disintegration is difficult to evaluate by plain light-microscopy in this circumstance.

Scanning electron microscopy (SEM) patently asserted membrane discontinuity and cell morphology. Basically, viably non-degenerated MCT cells were obviously round. Their cell membranes were completely integrated and covered by integral proteins on their external surfaces (Figure 7a,b). In contrast, for necrotized MCT cells, severe membrane interruption and membrane perforation were the predominant features of these cells. In addition, their surface proteins were rigorously collapsed or depleted (Figure 7b).

**Figure 8:**
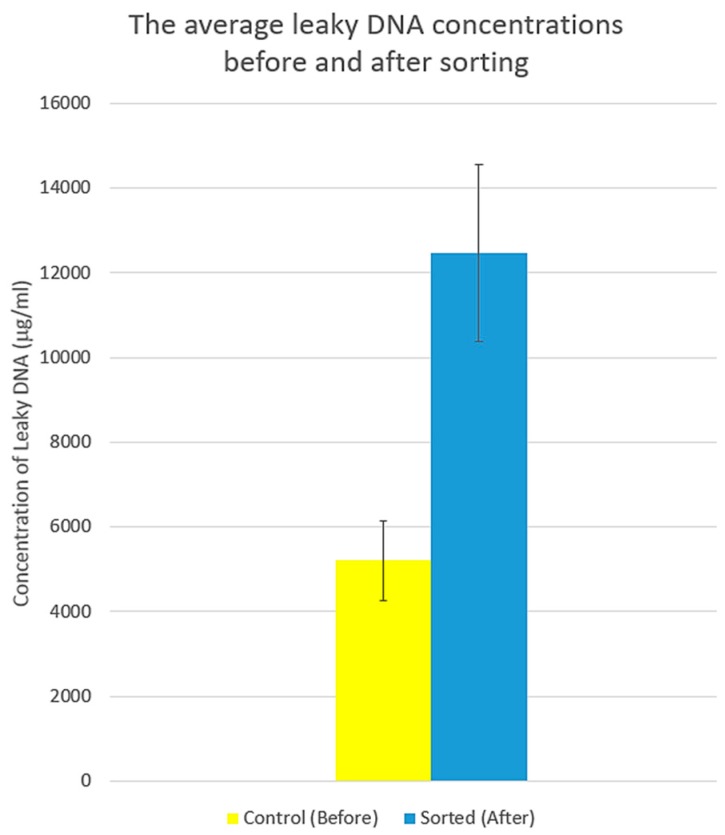
The histogram represents the average concentrations of leaky DNA in the supernatant of the control and sorted MCT specimens. The intensity of DNA leakage was approximately twofold increased after sorting, indicating membrane disintegration of the sorted MCT cells.

Spectrophotometrically, the measurement of leaky DNA in supernatant clearly implied cell membrane disintegration and cell wreckage. Before cell sorting, the mean concentration of leaky DNA was 5200 ± 928.72 μg/mL and its coefficient of variation (CV) was 17.85. However, it was approximately twofold after sorting: 12,470 ± 2080.41 μg/mL with the CV of 16.68 (Figure 8).

**Figure 9:**
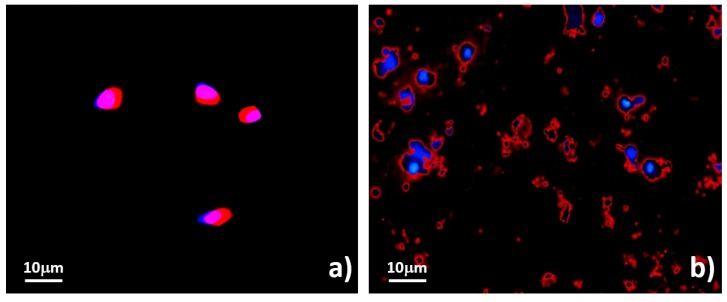
The functional viability of unsorted and sorted MCT cells with TMRM. (**a**) Unsorted MCT cells were positive for TMRM, of which the fluorescent signals were observed throughout the cytoplasm of the cells. Moreover, the method could clearly verify the morphology of both normal and necrotic MCT cells. (**b**) TMRM fluorescent signals were mainly positive at the perimeter of the cells. In addition, most sorted cells had erratic configurations.

Ultimately, with the lipophilic cation TMRM fluorophores, the viability probed by the functionality of the sorted MCT was assessed. Distinctly, the fluorescent signals were positive throughout the cytoplasm in viably unsorted MCT cells, indicating that most mitochondria were actively functioning (Figure 9a). Accordingly, their membrane potentials (ΔΨ_m_) were stabilized by proton transfers between their matrices and intermembrane spaces in association with succinate and ADP [47,48,49]. Therefore, active mitochondria would be an extrapolating parameter for cell viability. On the other hand, for sorted MCT cells, which underwent irreversibly degenerative changes, TMRM positivity was remarkably positive at the membrane rims of the sorted cells. However, the intensity of fluorescent signals was sharply dimmed or reduced. Additionally, the decay rate of the signals was proportional to their membrane potentials [47]. The unstained areas between the nuclear borders and the membrane rims would suggest inactively degenerated mitochondria (Figure 9b). Noticeably, the staining system could represent the conformation of the sorted MCT cells as well. As seen before sorting, the contour of the neoplastic cells was obviously round to slightly oval (Figure 9a). Meanwhile, TMRM fluoroscopy strongly demonstrated the bizarre features of degenerative and necrotic MCT cells after sorting. Some MCT cells had lost their nuclei and were composed of only the cytoplasm. Many of the remaining nuclei of the sorted MCT cells were erratic, severely elongated, or torn. In addition, cytoplasmic spillage was dominantly observed throughout the background. It was characterized by tiny cytoplasmic droplets encompassed by cell membranes without in situ nuclei. They were always positive with TMRM at their rims. Notably, most of the sorted MCT cells with anomalous configurations had stringent decreases in their cytoplasmic contents, resulting in the decrement of the N/C ratio (Figure 9b).

## 4. Discussion

Indubitably, live cells always provide valuable information that aids our understanding of how cells maintain their homeostasis after receiving external and internal stimuli. Under the disease process, all abnormality initiates from an alteration or a dysfunction of cells and/or subcellular components. Therefore, any information on the intricate biology of diseasing cells is indispensable. The loss of viable cells incurs the wasting of critical information on the biological mechanisms underlying diseases. As well as being at the heart of our microfluidic sorting system, the viability of MCT cells is therefore the crucial parameter for determining the system’s efficacy. To encourage this presumption, we had utilized a panel of biomedical methods for assessing the viability of the sorted MCT cells systematically.

Fundamentally, mammalian cells are liable to: (1) degenerate or necrotize under high-grade hydrodynamic shear stresses according to the lack of cell walls [50]; and (2) contain numerous cytoplasmic contents. Rheologically, there is a plethora of evidence showing that there are at least two well-known hydrodynamic components: fluid shear stress (FSS) and extensional flow stress (EFS), which govern the viability of mammalian cells under inertial hydrodynamic regimes.

For instance, the high-level fluid shear stress in a bioreactor can cause the sloughing of cultivated cells from their bases resulting in cell degeneration and necrosis [51]. In addition, hemolysis could come about when erythrocytes have flown through an area of high-grade extensional flow stress, such as a stenotic cardiac valve (20–50 Pa), an artificial heart and valve (1–1000 Pa), or a corporeal circulating device [52]. Fluorescence-activated cell sorting (FACS) itself can give rise to a noxious impact on sorted individual cells as well. It is due to high fluid shear stresses in the sheet-flow system [53]. The other examples are the deformation of endothelial cells induced by hemodynamic wall shear stress leading to atherosclerosis [54], atherothrombosis, and/or myocardial infarction under a turbulent flow [55].

Our postulation in this study was that both FSS and EFS would take place in our ASM sorting system. Intuitively, our CFD simulations substantially indicated the formation of FSS at the upper and lower boundaries of the spiral microchannel. Meanwhile, EFS was simultaneously observed at the lesser curvature of the microchannel. With respect to the study consequence, the hemocytometry enumeration ratified the loss of MCT cells in the system. The result unveiled that the MCT cell concentration after sorting sharply dwindled as compared to that before sorting. Approximately forty percent of the cells vanished in the spiral microchannel itself. Taken together, the combined effect of EFS and FSS might originate in the spiral microchannel and affect the viability of the sorted MCT cells.

The pathogenesis of hydrodynamic shear stress-induced cell damage in passive microfluidics is currently unclear. However, to the best of our knowledge, fluid shear stress (FSS) and extensional flow stress (EFS) are the critically influential parameters involved in HSS cell injuries. Ordinarily, mammalian cells are physiologically able to adapt themselves to cope with many mechanical injuries, including hydrodynamic shear stresses, by altering their intracellular components, such as cytoskeletal proteins, and/or the expressions of metabolic and survival genes. Meanwhile, under several pathological conditions, such as neoplasia, diseasing cells may lose these adaptive abilities and become ready to degenerate or necrotize.

Owing to this study, this consequence could explain some of the pathogenesis of HSS-induced cell injury. The first consideration is the competence of hydrodynamic shear stresses to induce the reorganization or disarrangement of cytoskeletons suddenly. This is a common cause of cellular deformity. Nevertheless, some deformed cells can recover spontaneously if they have not been across the point of no return [56,57]. In accordance with the morphological investigation, most deformed MCT cells were remarkably elongated, causing the decrement of average CMI. Doubtlessly, hydrodynamic shear stresses in the sorting system were the major detrimental factors that tampered with cytoskeletal resilience. In this case, the tensile strength of the cytoskeletal proteins would reach the maximal threshold and overcome the elastic recoil. Then, deformed cells could not retract to their original conformations. However, these cytoskeletons would not tear off at this stage. In contrast, most of the malignantly degenerated (necrotized) MCT cells had anomalous configurations. In this case, the hydrodynamic shear stresses (HSS) combinatorically overcame the tensile strength, resulting in mechanically denatured and ruptured cytoskeletons. Ultimately, necrotic MCT cells after sorting could not reshape themselves and were ripped off. Hence, cell wreckage or debris was the common feature observed in the scene as ratified by light microscopy. In addition, their average CMI was unmeasurable.

Probably, the secondary pathologic explanation is that of the disintegration of plasma membranes. Basically, cell membranes are composed of phospholipid bilayer domains. The continuity of cell membranes is maintained by the inherited hydrophobic bonds, referred as Van der Waals forces, of nearby long-chain fatty acids in the phospholipids, hydrogen bonds, and electrostatic bonds originating in the charged amino acid residues of transmembrane proteins. Biophysically, the shear modulus of plasma membranes is low as a result of bilayer fluidity (4× 10^−3^ –10 × 10^−3^ N/m), and the membrane tension (T_m_), a relative force applied to extend and deform the cell membranes of intact mammalian cells, is also small [58].

This implies that cell membranes are subject to be damaged by mechanical forces, i.e., hydrodynamic shear stresses. Relevant to our study result, scanning electron microscopy (SEM) explicitly unveiled lipid raft disintegration leading to membrane perforation in sorted MCT cells. Furthermore, the devastation of basic membrane components, including high-molecular-weight (HMW) integral protein, was an outstanding feature as well. Membrane perforation inevitably predisposed to cells to an increased permeant of water and trypan blue influx, followed by cell swelling, metabolic disturbances, and cytoplasmic protein staining with trypan blue. Furthermore, the efflux of macromolecules, such as DNA, in spite of the membrane defect was also observed. Hence, leaky DNA was a crucial investigative parameter. Normally, it relates to not only membrane perforation but also cell wreckage. As in our MCT case, the leaky DNA was increased by approximately two-fold when compared to the positive control.

The other influential factors that should be considered are the magnitude and the exposure time of both hydrodynamic shear stresses [59]. For example, Park and his coworkers have reported that human prostate cancer cells (PC 3) adhered on the PDMS floor of their microfluidic culturing devices were dead after experiencing a hydrodynamic wall shear stress over 0.5 Pa; however, at the lower magnitude of 0.2 Pa, the cells just became deformed [60]. Similarly, baby hamster kidney cells (BHK 21 c13) initiated cell necrosis when exposed to FSS at 1.5 Pa for 3 h in an adherent bioreactor. Moreover, these cells lost their biomembrane integrity, evidenced by the leakage of the internal cytoplasmic enzyme lactate dehydrogenase (LDH), at an FSS of 0.16 Pa [50].

Nevertheless, in one other study, colorectal adenocarcinoma cells (COLO 205) had been injected out of a syringe through the needle, and they were dead in the system owing to the tremendously high FSS of 75–600 Pa. Undoubtedly, at these FSS scales, the cells could not endure the shear stresses and then underwent cell necrosis [59,61]. Basically, there is an approximation that the threshold of shear stress for normal mammalian cells is in the range of 150 to 400 Pa [52]. However, in our sorting system, the time of exposure might be ignored because MCT cells had moved through the areas of hydrodynamic shear stresses ephemerally. Therefore, the majority should be due to the magnitude of HSS instead.

Last but not least, a cell species itself also involves hydrodynamic cell death. Fundamentally, the type of cell is the first determinant of cellular resistance to FSS and EFS at different levels. In our preliminary study, the results have suggested that canine leukocytes were much less vulnerable than MCT cells under the same sorting procedure (unpublished data). The survival rate of canine leukocytes was much higher when compared to MCT cells (90% and 40%, respectively). One of the probable explanations is the differences in the extracellular and intracellular components of the structural macromolecules or the competence of physiological adaptations between these two cell species.

Physiologically, most canine leukocytes usually circulate throughout the body via the circulatory system. Therefore, they always experience the mechanical effects of hemodynamic shear stress along their circulative trajectories. Pathologically, the primary function of leukocytes is to respond to the protective mechanism of the body referred to as inflammation. By this process, they usually deployed into the injured sites via a biological process called transmigration [57]. They have to emigrate through the tiny gaps between two adjacent endothelial cells of capillaries using pseudopods. After diapedesis, leukocytes glide on the subcutaneous connective tissues to the inflammatory sites. Because of their functionalities, canine leukocytes can remodel their structural components, especially some basic cytoskeletal proteins such as actin and myosin, for strengthening themselves. Undoubtedly, they can cope with the influence of the stresses greater than MCT cells.

However, in case of MCT cells, they are cutaneous cancer cells and are not motile. In addition, MCT cells usually release physiological chemomediators, especially vasoactive amines, from their cytoplasmic granules that are akin to normal mast cells. Hence, they do not need to fortify their structures similarly to canine leukocytes. Accordingly, MCT cells are less durable to high-grade hydrodynamic stresses than canine leukocytes, although they also experience low-intensity interstitial fluid shears in their microenvironments.

## 5. Recapitulation

We have herein reported the tenor of hydrodynamic cell injury in an MCT model after exposure two major hydrodynamic shear stresses (HSS): fluid shear stress (FSS) and extensional shear stress (EFS) that had formed in our predesigned spiral microchannel. Based on the study, CFD clearly verified the originations of FSS and EFS in our microdevice. Furthermore, the panel of systematic biomedical assays substantially indicated the decrement of cell viability and the increment of cell degeneration, necrosis, and/or debris of MCT cells after sorting. A hemocytometry enumeration confirmed a decreased MCT cell concentration as well.

The first possible pathomechanism underlying HSS-induced cell injury in our MCT model might be the sudden disorganization of cytoskeletons causing the morphological change in degenerated and necrotized cells as observed by light microscopy and CMI measurement. The second was the plasma membrane disintegration evaluated with SEM. Moreover, it led to reductions in cell susceptibility to the trypan blue exclusion assay and the leakage of DNA. TMRM also suggested the abating of mitochondrial functionality in both degenerative and necrotic MCT cells. Moreover, the other influential factor would be the type of cell itself. Therefore, hydrodynamic shear stresses in a spiral microchannel would have a noxious impact on the viability of the sorted cells, at least in our MCT model.

To the best of our knowledge, this study might be a pioneer work that provides preliminary information on the combinatorically detrimental effect of FSS and EFS on the viability of neoplastic cells after size-based cell sorting with a spiral microchannel. The pros of this study are a provision of a caveat on the next geometrical design of a curvilinear microchannel, which could mitigate the deadly effect of both de novo hydrodynamic shear stresses on sorted cells. In addition, the other biologically influential factors of neoplastic cells that might be involved in HSS-induced cell injuries, such as a variety of cell types and cell stages as well as HSS generation in delivery (feeding) systems, also require further investigations to complete our comprehension of hydrodynamic cell injury under inertial microfluidic cell sorting before the method will be in use in the upcoming years.
